# Quantitative analysis of T cell subsets in a population of Black women with invasive breast cancer

**DOI:** 10.1038/s41523-025-00780-5

**Published:** 2025-07-01

**Authors:** Angela R. Omilian, Lucas Mendicino, Anthony George, Tina Darabnoushtehrani, Rochelle Payne Ondracek, Wiam Bshara, Chi-Chen Hong, Bo Qin, Elisa V. Bandera, Thaer Khoury, Rikki Cannioto, Song Yao, Christine B. Ambrosone

**Affiliations:** 1 Department of Cancer Prevention and Control, Roswell Park Comprehensive Cancer Center, Buffalo, NY USA; 2 Department of Pathology, Roswell Park Comprehensive Cancer Center, Buffalo, NY USA; 3 Department of Biostatistics and Epidemiology, Rutgers School of Public Health, Piscataway, NJ USA; 4 Cancer Epidemiology and Health Outcomes, Rutgers Cancer Institute, New Brunswick, NJ USA

**Keywords:** Breast cancer, Cancer epidemiology, Cancer microenvironment, Tumour immunology

## Abstract

We compared T cell subpopulations in primary invasive breast tumors from Black and White women and investigated breast cancer subtype-specific associations of T cell abundance with survival in Black women. Multispectral immune staining was used to quantify helper, cytotoxic, and regulatory T cells in the tumor and stromal compartments of breast tissues. In fully adjusted models, breast tumors from Black women were significantly more likely than those from White women to have a higher abundance of cytotoxic T cells (IRR, 2.41; 95% CI, 1.43–4.05) and helper T cells (IRR, 1.80; 95% CI, 1.06–3.06), and these differences were more pronounced in the tumor than the stromal compartment. Among Black women, higher levels of T cells were associated with improved survival in women with triple-negative breast cancer, whereas a trend of poorer survival was observed in women with HER2-positive tumors. This study contributes to an accumulating body of evidence that the tumor-immune landscape differs between Black and White women.

## Introduction

Characterizing the tumor immune microenvironment (TIME) in diverse populations is essential for understanding mechanisms of cancer progression and immunotherapy response that may vary across different population groups. Immune cells interact with the host tumor to affect the course of disease and are associated with prognosis and treatment response in many types of cancer^1^. A growing body of evidence suggests that the abundance and composition of tumor-infiltrating immune cells varies between Black and White women^2–8^, but the precise quantitation of the specific immune cell subsets underlying these differences, their localization within the TIME, and how they impact survival outcomes is much less understood in Black women with breast cancer.

Careful annotation of the breast immune landscape in populations of Black women is needed, as most prior work on this topic has been largely confined to populations of predominantly White women. Research in well-characterized populations in the context of relevant covariates, as well as stratified by breast cancer subtype, is essential for improving our understanding of how immune infiltrates impact outcomes in breast cancer, especially for Black women. In the current study, we employed multispectral staining to delineate and quantify three T cell subpopulations—cytotoxic T cells, helper T cells, and regulatory T cells—in the Women’s Circle of Health Study (WCHS) and the Women’s Circle of Health Follow-up Study (WCHFS). For this population of Black and White women with invasive breast cancer, we compared the abundance of T cell subsets in the tumor and stromal compartments of the TIME and estimated associations of the abundance of T cell subsets with survival in Black women.

## Results

### Study population characteristics

Cohort characteristics are shown in Table 1, and the diagram of participant inclusion criteria is shown in Supplementary Fig. 1. In total, there were 490 women with invasive breast cancer (394 Black and 96 White). Compared with White women, Black women were significantly more likely to have triple-negative breast cancer (24.1% vs 9.4%, *p* = 0.005), have tumors that were higher grade, (54.0% grade 3 vs 35.5%, *p* = 0.004), and they were more likely to have received radiation therapy (70.7% vs 47.3%, *p* < 0.001). Black women were also more likely to have higher BMI (30.6 vs 26.9 kg/m^2^, *p* < 0.001) than White women. There were no significant differences in age, tumor stage, tumor size, lymph node status, or the receipt of surgery, chemotherapy, and hormone therapy between Black and White women in this study.

**Table 1:** Tumor and clinical characteristics of women with invasive breast cancer enrolled in the WCHS and WCHFS according to self-identified race (*N* = 490)

		Black	White	Overall	*p* Value^a^
Overall	*N*	394 (80.4)	96 (19.6)	490 (100%)	
Age at diagnosis, years	Mean (SD)	53.54 (11.04)	53.10 (9.86)	53.46 (10.81)	0.517
BMI, kg/m^2^	Mean (SD)	31.70 (6.84)	28.21 (6.51)	31.01 (6.91)	**<0.001**
Age of tissue specimen, years	Mean (SD)	9.14 (3.09)	14.21 (1.17)	10.13 (3.47)	**<0.001**
Stage	I	171 (43.4%)	39 (41.1%)	210 (42.9%)	0.842
	II	173 (43.9%)	42 (44.2%)	215 (44.0%)	
	III/IV	50 (12.7%)	14 (14.7%)	64 (13.1%)	
Grade	1	44 (11.3%)	18 (19.4%)	62 (12.8%)	**0.004**
	2	136 (34.8%)	42 (45.2%)	178 (36.8%)	
	3	211 (54.0%)	33 (35.5%)	244 (50.4%)	
Subtype	Luminal	238 (61.0%)	67 (69.8%)	305 (62.8%)	**0.005**
	HER2+	58 (14.9%)	20 (20.8%)	78 (16.0%)	
	TNBC	94 (24.1%)	9 (9.4%)	103 (21.2%)	
ER status	ER+	270 (68.5%)	79 (82.3%)	349 (71.2%)	**0.008**
	ER−	124 (31.5%)	17 (17.7%)	141 (28.8%)	
HER2 status	HER2+	58 (14.8%)	20 (20.8%)	78 (16.0%)	0.151
	HER2−	333 (85.2%)	76 (79.2%)	409 (84.0%)	
Tumor size, cm	Mean (SD)	2.38 (1.63)	2.11 (1.32)	2.33 (1.58)	0.141
Lymph node status	Positive	153 (39.7%)	41 (43.2%)	194 (40.4%)	0.543
	Negative	232 (60.3%)	54 (56.8%)	286 (59.6%)	
Surgery	No	1 (0.3%)	1 (1.1%)	2 (0.4%)	0.274
	Yes	392 (99.7%)	94 (98.9%)	486 (99.6%)	
Chemotherapy	No	124 (31.6%)	26 (27.4%)	150 (30.7%)	0.428
	Yes	269 (68.4%)	69 (72.6%)	338 (69.3%)	
Radiation	No	115 (29.3%)	49 (52.7%)	164 (33.7%)	**<0.001**
	Yes	278 (70.7%)	44 (47.3%)	322 (66.3%)	
Hormone therapy	No	134 (34.2%)	34 (35.8%)	168 (34.5%)	0.768
	Yes	258 (65.8%)	61 (64.2%)	319 (65.5%)	

^a^Continuous variables were compared using the Mann–Whitney *U*-test; categorical variables were compared using the chi-square test.

*P*-values that are <0.05 are in boldface type.

### Staining validation and quality control

In total, 2,526,889 cells were phenotyped, including 104,501 cytotoxic T cells, 102,231 helper T cells, and 9222 regulatory T cells. For all immune subsets in our study, measures of cell density and percent positivity of cells were highly correlated (≥0.93, Supplementary Fig. 2). The correlation of CD8 marker abundance from conventional immunohistochemistry (IHC) and multispectral (mIHC) was 0.83 (*p* < 0.0001) (Supplementary Fig. 3). We observed a significant effect for tissue sample age, especially for the FOXP3 marker; this was mitigated by adding a term in our multivariable models to account for tissue age. There were no batch effects for tissue sections cut at different timepoints. Likewise, comparisons among tissue microarray (TMA) slides were also non-significant apart from FOXP3, which had more zero-inflation that influenced its distribution across TMA slides. Marker variability within TMA slide was higher than variability between TMA slides (Supplementary Fig. 4).

### T cell abundance, race, and tumor factors

**Fig. 1:**
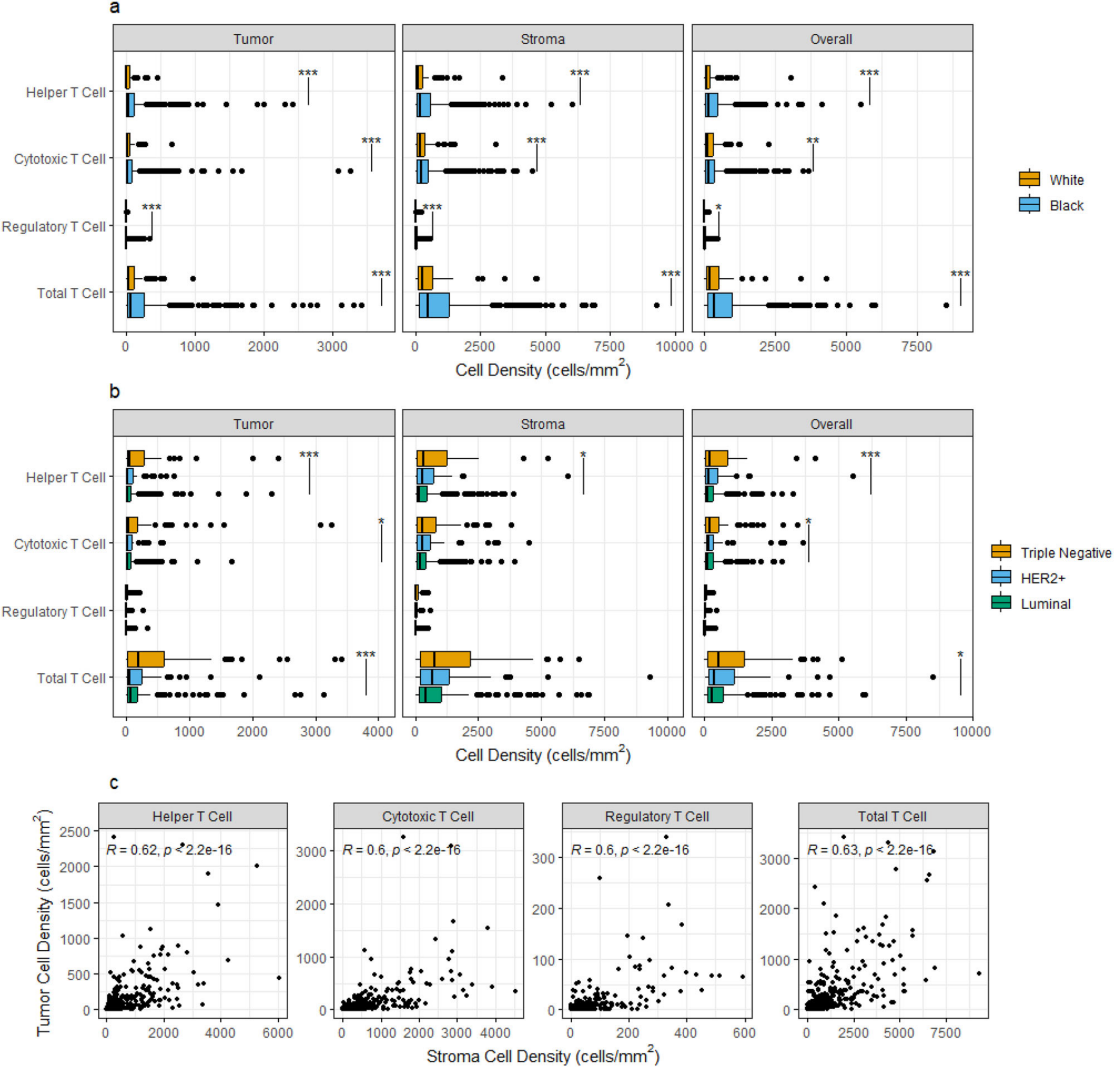
T cell subsets within the tumor, stromal, and overall (tumor + stroma) tissue areas. Breast tissues were segmented into tumor and stromal compartments for compartment-specific densities of T cell subpopulations. **a** Univariate comparisons of T cell densities between Black and White women. **b** T cell densities in the luminal, HER2-positive, and triple-negative breast cancer subtypes. **c** T cell densities in the tumor and stromal compartments were correlated. *p < 0.05, **p < 0.01, ***p < 0.001.

Of all cores analyzed, 91.8% contained helper T cells, 93.7% contained cytotoxic T cells, and 56.9% contained regulatory T cells. We detected 267 CD8^+^FOXP3^+^ immune cells, the majority of which were visually confirmed (Supplementary Fig. S5), but this population was too small for formal statistical analysis. Immune cell densities were higher in the stromal compartment compared to the tumor for all subsets (Fig. 1a). In univariate analyses, Black women had significantly higher levels of all three T cell populations in both the tumor and stromal compartments compared with White women. Helper T cells were the most prevalent in Black women, followed by cytotoxic T cells, and then regulatory T cells. For Black women, T cell populations were highest in women with triple-negative breast cancer (TNBC) and lowest in the luminal subtype with the exception of helper T cells in the tumor compartment, which were least abundant in human epidermal growth factor receptor 2 (HER2)-positive tumors (Fig. 1b). Immune cells were significantly correlated across the two tissue compartments (Fig. 1c). Univariate models revealed that the T cell populations in both the tumor and stromal compartments were significantly correlated with several tumor and clinical factors both when examining Black women (Table 2), and the overall cohort (Supplementary Table S2). For Black women, T cell densities showed trends of declining with age at diagnosis and were lower in overweight than in normal weight and obese patients. Variable patterns across the different T cell populations and tissue compartments were observed for AJCC stage, tumor size, and lymph node status, whereas increasing densities of T cells were observed with higher tumor grade (Table 2).

**Table 2:** Univariate negative binomial regression modeling of associations of T cell densities across levels of patient and tumor characteristics in Black women in the WCHS and WCHFS

			Helper T cell				Cytotoxic T cell				Regulatory T cell			
			Tumor		Stroma		Tumor		Stroma		Tumor		Stroma	
		*N*	Mean (SD)	*p* value	Mean (SD)	*p* value	Mean (SD)	*p* value	Mean (SD)	*p* value	Mean (SD)	*p* value	Mean (SD)	*p* value
Age at diagnosis	<40	39	245.71 (492.60)	0.26	994.40 (1327.00)	**0.008**	207.77 (500.62)	0.18	686.86 (833.85)	0.12	26.33 (59.11)	**0.001**	82.82 (127.75)	0.10
	40–50	100	143.28 (336.02)		466.52 (640.48)		115.41 (353.66)		489.07 (794.51)		7.87 (22.81)		35.66 (60.15)	
	50–60	133	116.30 (224.96)		536.45 (706.45)		110.30 (259.64)		483.89 (687.90)		11.15 (34.24)		57.00 (95.50)	
	60+	122	85.93 (166.49)		397.08 (711.84)		81.46 (184.68)		391.07 (603.50)		5.48 (14.39)		45.14 (88.09)	
BMI	<25	65	132.87 (291.96)	0.13	572.98 (825.13)	**0.002**	165.29 (439.14)	0.08	530.21 (799.76)	0.07	19.21 (48.58)	**<0.001**	66.52 (101.50)	**0.001**
	25–29.9	111	80.29 (144.17)		484.69 (920.63)		72.62 (191.34)		412.49 (696.45)		4.18 (12.72)		32.01 (71.54)	
	≥30	217	148.91 (327.19)		525.84 (708.17)		117.22 (293.94)		495.15 (690.24)		10.38 (30.38)		55.34 (94.00)	
Age of tissue specimen	<7 years	76	107.51 (192.43)	**<0.001**	593.44 (682.75)	**<0.001**	101.76 (249.10)	0.06	555.82 (870.53)	0.07	8.08 (16.82)	**<0.001**	72.38 (96.33)	**<0.001**
	7–9 years	195	163.32 (360.37)		571.12 (823.74)		105.38 (220.29)		498.90 (716.88)		15.04 (40.65)		62.93 (105.33)	
	10–13 years	67	92.43 (178.11)		496.57 (1005.01)		176.07 (544.45)		448.20 (618.62)		5.19 (18.93)		22.72 (41.79)	
	>13 years	56	65.16 (103.10)		276.54 (384.26)		74.53 (159.67)		325.08 (511.26)		1.26 (4.65)		10.55 (20.04)	
Stage	I	171	89.36 (164.61)	0.10	501.40 (753.87)	0.31	94.55 (286.89)	**0.047**	475.25 (689.35)	0.90	7.89 (23.78)	0.35	49.82 (83.20)	1.00
	II	173	160.38 (338.88)		590.97 (891.49)		141.49 (344.17)		520.51 (791.78)		9.76 (24.96)		51.66 (96.85)	
	III/IV	50	136.70 (362.30)		344.98 (449.15)		72.14 (124.31)		328.93 (405.29)		18.55 (58.88)		48.57 (90.62)	
Grade	1	44	95.72 (213.00)	**0.020**	377.24 (598.86)	0.46	64.48 (117.00)	0.21	334.23 (469.78)	0.07	6.91 (24.99)	**0.033**	32.14 (55.96)	0.39
	2	136	70.70 (160.96)		396.18 (589.42)		64.52 (180.58)		372.45 (626.71)		9.31 (37.88)		40.73 (86.70)	
	3	211	170.62 (344.10)		637.96 (917.39)		154.49 (375.43)		578.58 (788.59)		11.36 (27.25)		61.23 (97.22)	
Subtype	Luminal	238	103.06 (261.00)	0.20	450.78 (704.06)	**0.019**	76.08 (171.31)	0.27	411.40 (613.00)	0.07	7.29 (26.66)	**0.004**	40.50 (79.95)	**0.002**
	HER2+	58	96.68 (166.01)		545.89 (889.64)		82.69 (131.93)		564.38 (895.22)		10.51 (37.10)		55.09 (94.42)	
	TNBC	94	200.94 (366.40)		687.19 (919.93)		221.18 (526.80)		590.41 (797.81)		15.76 (34.15)		72.16 (106.51)	
ER	ER+	270	102.02 (252.94)	0.27	459.00 (691.86)	0.35	76.75 (166.54)	0.09	431.55 (661.24)	0.41	8.20 (30.67)	0.47	43.92 (85.71)	**0.039**
	ER−	124	179.96 (331.34)		655.60 (961.08)		189.77 (467.29)		574.56 (800.06)		14.12 (31.44)		64.73 (97.95)	
HER2	HER2+	58	96.68 (166.01)	0.07	545.89 (889.64)	**0.005**	82.69 (131.93)	0.23	564.38 (895.22)	**0.023**	10.51 (37.10)	**<0.001**	55.09 (94.42)	**0.008**
	HER2−	333	130.47 (297.01)		516.61 (776.32)		116.83 (320.94)		460.71 (673.56)		9.66 (29.13)		49.32 (89.17)	
Tumor size	<1	37	84.91 (162.67)	0.15	575.96 (1042.92)	**0.041**	51.46 (77.10)	0.19	488.60 (809.68)	0.07	13.60 (44.09)	**0.002**	56.65 (79.83)	0.14
	1–2	150	107.35 (204.21)		520.64 (692.78)		118.16 (332.74)		489.82 (647.38)		7.53 (17.34)		49.90 (87.54)	
	2+	205	149.32 (341.62)		516.12 (811.51)		119.99 (301.30)		468.87 (739.71)		11.38 (35.76)		50.26 (94.33)	
Lymph node status	Positive	153	150.10 (339.45)	0.55	537.50 (766.01)	0.95	103.75 (220.76)	0.13	477.74 (711.65)	0.22	13.19 (38.94)	0.73	62.17 (111.09)	0.056
	Negative	232	112.12 (239.96)		515.63 (818.18)		120.48 (347.39)		483.74 (719.47)		8.20 (24.95)		44.15 (74.07)	

*P*-values <0.05 are in boldface type.

**Fig. 2:**
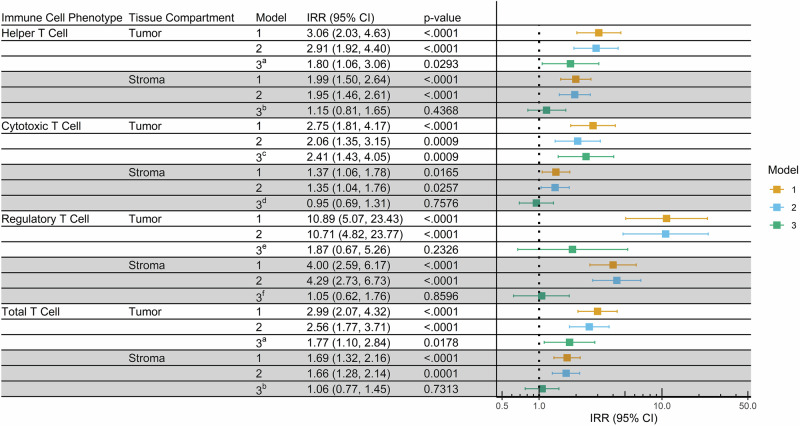
Models representing relative T cell densities in Black vs White breast cancer patients enrolled in the WCH studies (*N* = 490). Multivariate negative binomial regression modeling was used to estimate associations between immune cell densities and race. Model 1: adjusted for age at diagnosis. Model 2: age at diagnosis, subtype and grade. Model 3: age at diagnosis, tissue sample age, plus additional covariates determined by model fitting ^a^grade and tumor size, ^b^body mass index (BMI), grade, and subtype, ^c^tumor size, ^d^grade, ^e^BMI and subtype, ^f^grade and subtype. White women are represented by the vertical dotted line.

Multivariable negative binomial regression models representing relative T cell densities in Black vs White participants are shown in Fig. 2. In models adjusted for age (Model 1), there were significant differences between Black and White women for all three T cell subpopulations in both the tumor and stromal compartments. These differences remained after additional adjustment for subtype and grade (Model 2), with Black women having an incidence rate ratio (IRR) that was more than double that of White women in the tumor compartment. Covariates for Model 3 were determined by model fitting and varied by T cell subpopulation and tissue compartment (Fig. 2). Here, associations were attenuated but remained significant for helper T (IRR, 1.80; 95% confidence interval (CI), 1.06–3.06) and cytotoxic T cells (IRR, 2.41; 95% CI, 1.43–4.05) in the tumor compartment. However, in the stromal compartment, densities of T cells were not significantly different between Black and White women. In sensitivity analysis that compared Black and White women with similar tissue sample ages (87 Black and 96 White women diagnosed between 2003–2009), significant differences in the abundance of both helper T cells and cytotoxic T cells remained for Model 1 but not for Models 2 and 3 (Supplementary Table S3).

### T cell subsets and overall survival

**Fig. 3:**
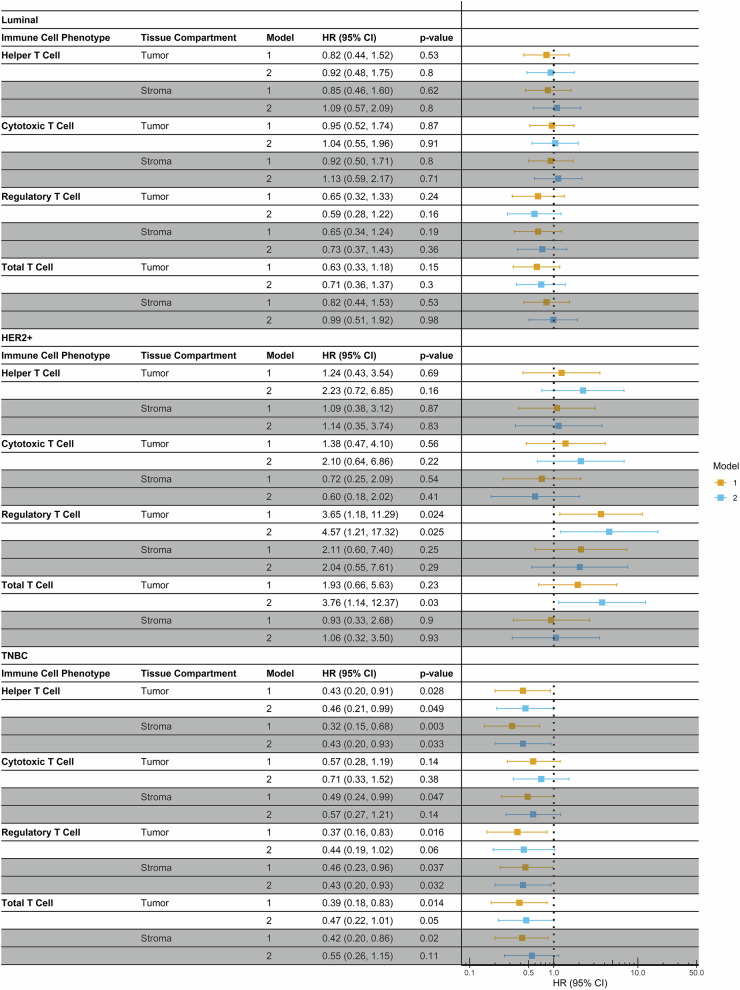
Associations of T cell subpopulations with overall survival in Black women enrolled in the WCHS and WCHFS (*N* = 386), stratified by breast cancer subtype. Model 1 is adjusted for age. Model 2 is additionally adjusted for body mass index (BMI), chemotherapy, grade, stage, and tumor size for the luminal subgroup; BMI, stage, and tumor size for the HER2-positive subgroup; and BMI and stage for triple-negative breast cancer.

Data for survival analysis were available for 386 Black women, with 90 deaths and a median follow-up of 9.2 years (interquartile range (IQR): 7.02–10.92). Models depicting associations of T cell subpopulations with overall survival in Black women are shown in Fig. 3. For the luminal subgroup, there were no significant associations between T cell subsets and overall survival in either the tumor or stromal compartments. We did observe significant associations with HER2-positive tumors, but only in the tumor compartment, not in the stroma. Here, increased densities of regulatory T cells and total T cells were associated with poorer survival outcomes. In fully adjusted models, Black women with HER2-positive tumors with higher levels of regulatory T cells had more than four times the hazard of death than those with lower levels of regulatory T cells (hazard ratio (HR) = 4.57; 95% CI, 1.21–17.32). In TNBC, we observed that higher levels of all three T cell subpopulations were associated with improved survival. Higher levels of helper T cells in both the tumor and the stroma were significantly associated with more than a twofold reduction in the hazard of death (tumor: HR = 0.46; 95% CI, 0.21–0.99; stroma: HR = 0.43; 95% CI, 0.20-0.93). There were similar relationships for regulatory T cells, which were significant in the stroma (HR = 0.43, 95% CI, 0.20–0.93), but not significant in the tumor compartment (HR = 0.44; 95% CI, 0.19–1.02).

## Discussion

In these multispectral analyses of T cell subsets and with fully adjusted statistical models, we found that breast tumors from Black women had an increased abundance of cytotoxic and helper T cells compared with White women, and these differences were more pronounced in the tumor than the stromal compartment. Variation across the tumor and stromal compartments may be an important feature of differences in breast tumor biology between Black and White women, a finding enabled by the multispectral staining technology that facilitates analysis of immune cells in their tissue compartment-specific spatial context. Consistent with our findings, prior studies have similarly reported that attributes of the immune infiltrate in the tumor compartment may not mirror those of the stroma in the breast TIME^9–12^.

These results reinforce prior reports of breast tumors that document higher levels of immune infiltration and inflammatory markers in Black women. While there are exceptions^13,14^, indicators of a more robust immune response in Black women has emerged across several studies that collectively employ a wide variety of detection methods, including immunostaining, gene expression, methylation, and mutational analyses of breast tumors^2–8,15–19^. Furthermore, Black women have higher levels of circulating inflammatory biomarkers and cytokines that are associated with breast cancer risk and survival^20,21^. Previous studies of gene expression have shown that Black women and women of African ancestry are more likely to have TIMEs with features of T cell exhaustion and downregulation of immune cell activation^5,22^, suggesting qualitative differences in the composition of the immune infiltrate. Collectively, these data suggest a role for immunity in the unfavorable outcomes experienced by Black women with breast cancer that could potentially inform therapeutic approaches specific to this population. As most cancer therapeutics have been built upon research conducted in White populations with European ancestry, our results augment the existing body of evidence signifying the need to include diverse populations in oncology research. At present, we do not know the underlying reasons, whether biological or social, for why Black women have higher abundances of certain immune cell populations, and this is a topic for future investigation.

T cell abundance in relation to breast cancer outcomes has been studied thoroughly in White women, in which higher levels of stromal tumor-infiltrating lymphocytes (TILs) are commonly associated with favorable outcomes and therapy response, especially in HER2-positive and triple-negative tumors^23–27^. However, fewer studies have reported specifically for Black women. Restricting analyses of survival to the WCHS Black population only, we observed distinct patterns by breast cancer subtype, and a trend of worse survival with increasing abundance of immune cells for the HER2-positive subgroup. Here, there were poorer survival outcomes when T cell populations were localized to the tumor compartment compared to the stroma, and the associations were significant for regulatory T cells and total T cells in the tumor compartment. This may be a spurious finding due to the small sample size of the HER2-positive group. Moreover, T cells were less abundant in the tumor compared with the stromal compartment, so the vagaries of sampling a small number of cells may have also contributed to this finding. These caveats are counterbalanced by the distinct and precise quantitation of immune cells in their spatial context that is afforded by the multispectral staining platform. As counts of stromal TILs are typically used to quantify immune infiltration, the possibility remains that our findings represent a unique aspect of tumor biology in Black women that may have been masked in earlier work that did not incorporate precise counts of immune cells within the tumor compartment.

To our knowledge, there are no published reports specific to Black women that investigate tumor compartment-specific counts of T cell subsets as well as their associations with survival outcomes in the HER2-positive subgroup. Our findings, if confirmed in larger studies, suggest unique attributes of the lymphocytic infiltrate in Black women with HER2-positive breast cancer. Newman and colleagues, with an analysis of RNA sequencing of TCGA samples, found that having high tumor-associated lymphocytes was associated with better survival in White but not in Black patients^28^. However, stratification by subtype was not performed, leaving unclear whether the finding we observed in WCHS may also be observed in TCGA. Likewise, a study of West African tumors showed that higher TIL frequencies had no significant influence on survival, but also did not examine the HER2-positive group specifically^19^. As subtype is a key feature of survival analyses, future investigations with sample sizes large enough to permit stratification by subtype and adjustment for relevant clinical factors are needed in large populations of Black women with breast cancer.

The prognostic value of T cells varied across the different subtypes of breast cancer. Within the TNBC group, we observed that higher densities of all three T cell subpopulations were associated with favorable survival in both the tumor and stromal compartments. In particular, regulatory T cells were associated with significantly improved survival in TNBC while simultaneously prognosticating worse survival in the HER2-positive subgroup. As regulatory T cells are known to suppress cytotoxic T cells and facilitate immune tolerance and homeostasis, they are often regarded as an immunosuppressive population that portends tumor progression and unfavorable outcomes. However, they occur secondarily after an active CD8^+^ T cell response, and their prognostic impact is nuanced and context-dependent^29,30^, potentially indicating a robust immune response in certain settings. In accordance with our findings, previous work has also shown that regulatory T cells or FOXP3^+^ cells are a favorable prognostic indicator, particularly in the estrogen receptor (ER)-negative and/or triple-negative subgroups^31–33^. Similarly, a recent study of an M2-like macrophage marker showed that increasing CD163^+^ immune cells were also associated with pronounced improvement in overall survival in TNBC, even though this marker is generally regarded as signifying a pro-tumor microenvironment. It is unclear whether qualitative or merely quantitative differences underlie the association of higher immune infiltration into the TIME and improved survival in the triple-negative subtype^27^. Future studies with expanded panels of markers that delineate functional status will be informative to determine if specific combinations of immune subsets are driving these associations.

There are some stark differences between measuring specific T cell populations with the multispectral technology vs stromal TILs on hematoxylin–eosin-stained slides. Measures of stromal TILs can be confounded by heterogeneity in their distribution, improper delineation of the tumor/stroma boundary, and the inadvertent inclusion of other inflammatory cells^34^. By and large, using the multispectral staining approach avoids these pitfalls and facilitates precise counts of specific cell phenotypes. Similarly, gene expression studies of bulk tumor samples also obfuscate tissue compartment-specific signals. Here, we observe additional value in enumerating T cell populations within the tumor and stromal compartments separately. As the spatial-relational effects of immune cells with the TIME are increasingly found to provide additional prognostic information in breast and other cancer types^9,35–38^, the detailed mapping of T cell populations within the TIME and the precise measurement of their distances to other T cells as well as malignant cells is an important next direction for this research.

Limitations of this work include that we used a single multiplexed panel that identified T cell subpopulations on a coarse scale; the technology that was available to us at the initiation of this study did not accommodate additional markers that could characterize at a finer scale the various functional states of T cells. Although whole sections are better for studies of the tumor-immune landscape, a study of this size is not feasible with whole sections, and thus TMAs were used as is commonly done in other large studies^39–41^. As we avoided the tumor margins when coring for TMA construction, our results do not apply to the tumor interface or other non-tumor regions. Changes in recruitment strategies over time in the WCHS led to differences in tissue sample age between Black and White women, potentially introducing bias due to differential preservation quality and staining variability. In our main analyses, we addressed this by including a term for tissue sample age in our statistical models. However, sensitivity analysis limited to tissue samples from the same timeframe showed significant differences in our age-adjusted models, but not the fully adjusted models. Thus, we cannot rule out the possibility that the differences in T cell abundance between Black and White women are due to differences in tissue age or other unmeasured factors relating to recruitment across different time periods. Lastly, as the WCHS was deliberately designed to characterize features of breast cancer in Black women, the generalizability of our findings is limited to this population. However, this population-based cohort of Black women is one of the largest to date with multispectral immune data that were quantified with a high degree of precision and analyzed with reference to comprehensive clinical variables, including more than nine years of follow-up.

In conclusion, Black women in the WCHS have significantly more cytotoxic and helper T cells in the tumor compartment than White women. As immunity is affected by social, environmental, and genetic factors, further studies that discern the causes of dissimilar immune profiles across diverse populations are needed. Regardless of the upstream causal factors for increased T cells in Black women with breast cancer, our study contributes to an accumulating body of evidence that the tumor-immune landscape is different in breast tumors from Black women. Future studies should further characterize the immune landscape in Black women, including more markers that can refine the T cell subsets and their functional states to potentially identify areas to remedy disparities in outcomes through targeted therapeutic interventions.

## Methods

### Patient population and tissue samples

The data and tumor specimens for this study were drawn from cases from the WCHS, a case–control study designed to examine risk factors for aggressive breast cancer in Black and White women, and the WCHFS, which expanded the scope of WCHS to include factors that affect survivorship and prognosis. Both studies have been described extensively in our previous work^13,42,43^. Briefly, participants were 20–75 years; self-identified as Black or White; had primary, histologically confirmed invasive breast cancer or ductal carcinoma in situ (DCIS); and had no previous history of cancer other than non-melanoma skin cancer. An in-depth at-home interview was conducted to collect data on risk factors for breast cancer, and cases signed a release for their formalin-fixed paraffin-embedded tumor blocks and medical records. All women provided informed consent, and the study protocol was approved by the Institutional Review Boards at Rutgers Cancer Institute and Roswell Park Comprehensive Cancer Center. As the WCHS and WCHFS focused on recruiting Black women, the number of cases from Black women in our dataset far exceeds the number of White women (Black: *N* = 394, White: *N* = 96). Thirty-four patients who received neoadjuvant therapy were excluded. Patient tissues for the Women’s Circle of Health studies are available in TMA format. TMAs were built previously under the guidance of a breast pathologist (T.K.). TMA cores ranged in size from 0.6 to 1.2 mm in diameter, and the majority of patient tumors in this study were represented by at least two TMA cores. At the time this study was initiated, cases diagnosed from 2003 to 2017 were built into TMAs.

### Quantification of T cell subpopulations in the tumor and stromal compartments

Multispectral IHC was performed by the Human Immune Monitoring Core at the Columbia University Medical Center. Antibody staining optimization was carried out on breast TMAs that included tonsil cores as controls for immune markers. TMAs were stained in a single batch to reduce technical variability using the OPAL 7-Color Kit (Akoya Biosciences, Marlborough, MA). The panel included CD4, CD8, FOXP3, ER alpha, and pan-cytokeratin and 4′,6-diamidino-2-phenylindole (DAPI) to differentiate tumor epithelium and cell nuclei, respectively. CD8 is a marker of cytotoxic T cells, and CD4^+^ helper T cells help to orchestrate the immune response against cancer, whereas regulatory T cells are likely to suppress antitumor T cell responses^44^. Total T cells, which included all three subpopulations (cytotoxic, helper, and regulatory T cells) added together, were also evaluated. Antibody and staining information are shown in Supplementary Table 1.

TMA cores were individually scanned with the Vectra Polaris Automated Quantitative Digital Pathology System and analyzed with the inForm software (v2.7.1, Akoya Biosciences). Each core image was spectrally unmixed and visually inspected by a pathologist for quality and to confirm that each TMA core contained invasive breast tumor. TMA cores that contained predominantly normal tissue or DCIS were excluded. Cores were excluded if the tissue morphological structure was damaged by the iterative staining process, i.e., cores with large breaks or holes. Additionally, cores were visually inspected for tissue regions that could obfuscate automated image analyses, like folded tissue or other artifacts; if present, these regions were excluded with the negative pen tool. One thousand one hundred and forty-seven cores representing 490 cases were retained for analysis.

Automated segmentation of the tissue into tumor and stroma compartments was performed using pan-cytokeratin as a marker of tumor epithelia and training an algorithm to identify each tissue compartment. For cell segmentation, DAPI was used to define the nucleus for each cell with assistance from CD8 to define the cell membrane. Initially, 7–10 cells for each marker were manually selected to train the cell phenotyping algorithm, and then images were visually inspected by a pathologist (W.B.) and the algorithm refined accordingly. Each core image was digitally scored for the percent and density (per square micron of tissue) of positive cells for each marker both individually and in combinations that signify immune cell subsets: helper T cell (CD4^+^/CD8^−^/FOXP3^−^), cytotoxic T cell (CD4^−^/CD8^+^/FOXP3^−^), and regulatory T cell (CD4^+^/CD8^−^/FOXP3^+^). For participants with multiple cores, cell density was calculated by dividing the total number of positive cells for a given immune subset across all cores by the total tissue area of those cores.

### Quality control measures for mIHC

For the CD8 marker, mIHC staining was validated by comparing to conventional IHC of the same tissues from a previous study^4^. For each marker, we investigated several potential sources of technical variation, including TMA slide, TMA section cut date, and tissue sample age. To visualize immune cell marker variation within and between groups, the raw and log-transformed cell densities were plotted. Group medians were compared using the Wilcoxon Rank Sum and Kruskal–Wallis tests. Due to the highly variable, over-dispersed nature of the data, intraclass correlation coefficients were calculated using generalized linear mixed models with a negative binomial distribution to determine how much of the variability in immune cell density was attributed to differences in TMA slides or tissue sample age. Finally, influence statistics (Cook’s D, DFBetas) and Tukey’s methods were used to identify specific TMA slides and cores that may be influential or possible candidates for exclusion that were then visually inspected with assistance from our study pathologists.

### Epidemiological, tumor, and outcome variables

Women self-identified their race in the baseline questionnaire. Tumor and clinicopathological factors were abstracted from the patient pathology report, medical records, or obtained through linkage with the New Jersey State Cancer Registry. Tumor and clinical variables included AJCC stage, grade, tumor size, node status, and treatment (surgery, chemotherapy, radiation therapy, and/or hormone therapy). Breast cancer subtypes were inferred from ER, progesterone receptor (PR), and HER2 status information from the pathology reports as follows: luminal (HR+/HER2−), HER2-positive (HR+/HER2+ or HR−/HER2+), and triple-negative (HR−/HER2−), where hormone receptor (HR+) refers to ER+ and/or PR+. Other factors such as age and body mass index were obtained by interviewer- and self-administered questionnaires at baseline and/or have been previously described^45^. Data on vital status, including dates and causes of death, were ascertained through linkage with the New Jersey State Cancer Registry files for 386 Black women. Overall survival (OS) was the outcome of interest, and time to follow-up was calculated from the date of diagnosis until the date of last follow-up (August 2023) or death from any cause.

### Statistical analyses

Patient demographic and clinical factors were summarized using the median and IQR for continuous variables and relative frequencies for categorical variables. To model immune phenotypes as a function of patient and clinical tumor characteristics, negative binomial regression was used with an offset term for total cell density due to overdispersion observed in the immune data. Multivariable models to assess the associations between race and T cell density were formulated based on model fitting. We investigated three models—Model 1: adjusted for age at diagnosis; Model 2: age at diagnosis, subtype, and grade; Model 3: age at diagnosis, tissue sample age, plus additional covariates selected based on model fitting, tailored to the data distribution of each immune cell subset. Model assumptions were verified graphically, and IRRs and 95% CI conveyed the relative difference of T cell abundance in Black vs White women. To address differences in tissue specimen age between Black and White women, we adjusted for tissue sample age in statistical models and conducted sensitivity analyses.

To define associations of the different T cell subsets with OS in Black women, multivariable Cox regression models were used to compute HRs and 95% CIs. As there are differences in survival patterns by breast cancer subtype^46,47^, we stratified the cohort by subtype. Given the lack of consensus for what qualifies as high vs low T cell infiltration in the literature, T cell densities were categorized at the median for cytotoxic T cells and helper T cells, while regulatory T cells were categorized as present or absent in the tumor compartment, due to the smaller number of positive cells in this group. Variables that were significantly associated with T cell density and survival in the univariate setting were added to a multivariable model and sequentially removed while assessing model fit using a likelihood ratio test. Covariates were retained in the final model if their inclusion improved model fit and varied by breast cancer subtype and T cell subset. The proportional hazards assumption was verified graphically by analyzing log-log survival curves and scaled Schoenfeld residuals; no violations were observed. All statistical analyses were conducted in SAS (version 9.4) and two-sided *p* values ≤ 0.05 were considered statistically significant.

## Supplementary information

Omilian_SuppMaterials_5-28-25_npj

## Data Availability

Epidemiological data and image data are available from the corresponding author upon reasonable request.
