# Supplementary material for: Quantitative analysis of T cell subsets in a population of Black women with invasive breast cancer
Source: NPJ Breast Cancer. 2025 Jul 1;11:64. doi: 10.1038/s41523-025-00780-5 (PMC12218130; doi:10.1038/s41523-025-00780-5)
Supplement: Supplementary file 1 — Omilian_SuppMaterials_5-28-25_npj [file 41523_2025_780_MOESM1_ESM.pdf]

Supplemental Materials

Supplemental Tables

Table S1. Antibody and staining information

Antibody	Clone	Vendor	Catalog #	Antibody dilution	Opal
CD8	4B11	Leica	CD8-4B11-L-CE	1/100	520
FOXP3	236A/E7	Abcam	ab20034	1/150	620
CD4	EPR6855	Abcam	ab133616	1/500	570
ER-alpha	SP1	Cell Marque	249R-16-ASR	1/200	650
Pan-cytokeratin	AE1/AE3	BioLegend	914201	1/160	690

Table S2. Univariate negative binomial regression modeling of associations of T cell densities across levels of patient and tumor characteristics in the overall cohort (Black and White women) in the WCHS and WCHFS (N=490).

			Helper T Cell				Cytotoxic T Cell				Regulatory T Cell				Total T Cells			
			Tumor		Stroma		Tumor		Stroma		Tumor		Stroma		Tumor		Stroma	
		N	Mean (SD)	P-value	Mean (SD)	P-value	Mean (SD)	P-value	Mean (SD)	P-value	Mean (SD)	P-value	Mean (SD)	P-value	Mean (SD)	P-value	Mean (SD)	P-value
Age at diagnosis	< 40	47	215.08 (454.85)	0.31	892.13 (1245.86)	***0.020***	189.34 (465.24)	0.11	698.92 (861.84)	0.13	21.85 (54.65)	***<.001***	70.17 (119.69)	0.12	426.27 (736.26)	0.17	1661.22 (1973.99)	***0.046***
	40 - 50	129	119.58 (301.51)		437.33 (649.75)		97.53 (313.40)		450.48 (724.18)		6.35 (20.31)		30.83 (55.87)		223.46 (480.72)		918.64 (1291.83)	
	50 - 60	166	103.82 (207.95)		463.62 (657.95)		97.69 (235.62)		431.32 (630.47)		9.16 (30.93)		46.89 (88.08)		210.67 (402.64)		941.83 (1231.73)	
	60 +	148	76.70 (153.77)		361.03 (659.62)		74.77 (170.50)		376.69 (565.53)		4.72 (13.22)		39.94 (82.99)		156.19 (299.87)		777.66 (1182.66)	
Race	Black	394	126.55 (281.90)	***<.001***	520.88 (790.62)	***<.001***	112.32 (300.15)	***<.001***	476.55 (709.95)	***0.037***	10.07 (31.00)	***<.001***	50.47 (90.14)	***<.001***	248.93 (486.59)	***<.001***	1047.90 (1421.63)	***<.001***
	White	96	44.36 (86.69)		244.93 (453.76)		46.97 (85.78)		318.21 (430.76)		1.03 (3.25)		11.31 (31.98)		92.37 (150.74)		574.44 (830.45)	
BMI	< 25	103	97.92 (239.87)	0.12	459.10 (708.14)	***0.023***	123.54 (358.50)	0.17	470.23 (718.38)	0.09	12.30 (39.56)	***<.001***	46.30 (87.93)	***0.007***	233.76 (496.48)	0.15	975.64 (1269.40)	***0.024***
	25 - 29.9	135	77.29 (138.27)		465.65 (890.09)		67.99 (175.97)		399.73 (655.13)		3.55 (11.64)		28.22 (66.75)		148.84 (263.65)		893.60 (1454.29)	
	>= 30	251	133.86 (308.03)		472.17 (674.15)		106.96 (275.39)		461.55 (654.04)		9.24 (28.44)		49.37 (89.00)		250.06 (496.68)		983.08 (1308.29)	
Age of tissue specimen	< 7 years	76	107.51 (192.43)	***<.001***	593.44 (682.75)	***<.001***	101.76 (249.10)	***0.002***	555.82 (870.53)	***0.004***	8.08 (16.82)	***<.001***	72.38 (96.33)	***<.001***	217.36 (389.52)	***<.001***	1221.64 (1405.51)	***<.001***
	7 – 9 years	195	163.32 (360.37)		571.12 (823.74)		105.38 (220.29)		498.90 (716.88)		15.04 (40.65)		62.93 (105.33)		283.74 (519.06)		1132.95 (1500.90)	
	10 – 13 years	94	78.00 (156.25)		443.59 (921.90)		137.60 (463.52)		412.04 (556.78)		4.11 (16.15)		21.58 (43.22)		219.71 (533.95)		877.21 (1399.12)	
	> 13 years	125	54.15 (97.65)		244.57 (358.01)		60.36 (128.54)		320.40 (481.41)		1.05 (3.86)		9.36 (21.84)		115.57 (196.41)		574.34 (801.19)	
Stage	I	210	78.99 (154.52)	0.13	433.17 (701.58)	0.56	82.82 (261.08)	***0.048***	431.69 (641.97)	0.97	6.62 (21.67)	0.32	42.08 (77.14)	0.85	168.43 (345.53)	0.08	906.94 (1311.95)	0.87
	II	215	139.26 (309.64)		539.77 (848.75)		125.97 (314.04)		492.25 (751.79)		8.05 (22.69)		43.54 (89.24)		273.27 (534.36)		1075.57 (1483.26)	
	III / IV	64	118.61 (322.57)		339.27 (425.96)		66.99 (112.36)		339.02 (382.67)		14.76 (52.44)		43.30 (85.32)		200.36 (394.48)		721.59 (784.45)	
Grade	1	62	75.86 (185.68)	***0.005***	306.42 (531.08)	0.26	50.07 (101.35)	0.10	285.95 (407.21)	***0.046***	4.90 (21.22)	***0.047***	24.55 (48.88)	0.07	130.83 (290.22)	***0.043***	616.92 (940.78)	0.10
	2	178	60.85 (143.89)		358.70 (580.24)		59.91 (161.15)		354.24 (573.94)		7.31 (33.30)		32.37 (77.43)		128.07 (289.42)		745.32 (1090.77)	
	3	244	157.36 (324.54)		595.63 (874.16)		142.90 (353.00)		559.34 (765.60)		10.08 (25.59)		56.00 (93.18)		310.33 (547.90)		1210.97 (1543.00)	
Subtype	Luminal	305	89.00 (234.25)	0.38	405.01 (669.80)	***0.025***	70.29 (158.32)	0.30	391.94 (583.54)	0.07	5.85 (23.72)	***0.004***	33.26 (72.60)	***<.001***	165.14 (365.72)	0.21	830.21 (1233.04)	***0.022***
	HER2 +	78	78.80 (147.78)		479.56 (795.67)		72.75 (117.79)		517.82 (799.26)		8.22 (32.25)		46.96 (86.80)		159.77 (254.91)		1044.34 (1464.52)	
	TNBC	103	194.10 (354.60)		641.95 (891.70)		204.78 (505.98)		551.09 (773.64)		14.53 (32.87)		66.95 (103.36)		413.41 (665.10)		1259.99 (1503.05)	
ER	ER +	349	87.41 (226.21)	0.19	414.20 (655.84)	0.34	70.76 (153.24)	0.06	413.43 (622.37)	0.63	6.58 (27.18)	0.68	36.70 (78.22)	***0.018***	164.75 (355.27)	0.10	864.33 (1238.89)	0.33
	ER -	141	167.48 (316.39)		597.05 (918.29)		170.69 (441.25)		524.99 (763.47)		12.55 (29.79)		57.88 (93.90)		350.71 (595.85)		1179.92 (1542.16)	
HER2	HER2 +	78	78.80 (147.78)	0.19	479.56 (795.67)	***0.009***	72.75 (117.79)	0.35	517.82 (799.26)	***0.026***	8.22 (32.25)	***0.001***	46.96 (86.80)	***0.005***	159.77 (254.91)	0.13	1044.34 (1464.52)	***0.006***
	HER2 -	409	115.32 (272.81)		464.06 (737.49)		104.00 (293.46)		431.07 (639.20)		8.03 (26.53)		41.66 (82.56)		227.34 (471.14)		936.79 (1316.81)	
Tumor size	< 1	48	66.84 (146.42)	0.30	453.28 (940.86)	0.29	47.65 (75.23)	0.30	430.84 (731.64)	0.29	10.64 (38.99)	***0.002***	44.21 (73.59)	0.31	125.13 (201.69)	0.20	928.33 (1612.06)	0.39
	1 - 2	189	94.47 (188.53)		452.92 (643.54)		102.50 (299.16)		440.52 (597.98)		6.19 (15.74)		41.39 (80.11)		203.16 (426.45)		934.84 (1220.30)	
	2 +	249	132.70 (314.10)		487.24 (779.43)		108.70 (277.93)		458.27 (708.89)		9.58 (32.70)		44.26 (88.43)		250.98 (492.30)		989.78 (1378.85)	
Lymph node status	Positive	194	127.52 (305.79)	0.40	472.43 (703.84)	0.94	92.39 (199.42)	0.08	435.71 (647.90)	0.40	10.58 (34.94)	0.77	51.40 (102.20)	***0.049***	230.49 (453.85)	0.20	959.54 (1291.71)	0.61
	Negative	286	99.29 (222.03)		466.69 (780.84)		105.96 (317.09)		457.25 (688.62)		6.88 (22.69)		38.00 (68.95)		212.13 (445.96)		961.95 (1387.82)	

Table S3. Sensitivity analyses for relative T cell densities in Black versus White breast cancer patients enrolled in the WCH studies.

Models representing relative T cell densities in Black versus White breast cancer patients enrolled in the WCH studies from 2003-2009 (N=183). Multivariate negative binomial regression modeling was used to estimate associations between immune cell densities and race. Model 1: adjusted for age at diagnosis. Model 2: age at diagnosis, subtype and grade. Model 3: age at diagnosis, subtype, grade, and tissue sample age. Incidence rate ratios (IRR) and Chi-square p-values are reported.

N (Total) = 183<br>N (B) = 87<br>N (W) = 96			**Helper T Cells**				**Cytotoxic T cells**				**Regulatory T cells**				**Total T cells**			
			**Tumor**		**Stroma**		**Tumor**		**Stroma**		**Tumor**		**Stroma**		**Tumor**		**Stroma**	
			IRR	p-value	IRR	p-value	IRR	p-value	IRR	p-value	IRR	p-value	IRR	p-value	IRR	p-value	IRR	p-value
Model 1	Race	White	Ref.	0.047	Ref.	0.236	Ref.	0.023	Ref.	0.970	Ref.	0.280	Ref.	0.978	Ref.	0.015	Ref.	0.487
		Black	1.86 (1.01, 3.45)		1.29 (0.85, 1.95)		1.92 (1.09, 3.37)		1.01 (0.71, 1.42)		2.13 (0.54, 8.39)		1.01 (0.48, 2.11)		1.90 (1.13, 3.19)		1.13 (0.80, 1.59)	
Model 2	Race	White	Ref.	0.466	Ref.	0.787	Ref.	0.484	Ref.	0.547	Ref.	0.704	Ref.	0.586	Ref.	0.478	Ref.	0.834
		Black	1.27 (0.67, 2.42)		1.07 (0.67, 1.69)		1.26 (0.66, 2.39)		0.89 (0.61, 1.30)		0.70 (0.12, 4.31)		0.80 (0.46, 1.77)		1.23 (0.70, 2.15)		0.96 (0.66, 1.40)	
Model 3	Race	White	Ref.	0.533	Ref.	0.710	Ref.	0.570	Ref.	0.544	Ref.	0.408	Ref.	0.640	Ref.	0.581	Ref.	0.875
		Black	1.23 (0.65, 2.32)		1.09 (0.69, 1.74)		1.21 (0.63, 2.32)		0.89 (0.61, 1.30)		0.43 (0.06, 3.12)		0.83 (0.37, 1.84)		1.17 (0.67, 2.06)		0.97 0.66, 1.42)	

Supplemental Figures

Figure S1. Inclusion criteria for identifying eligible WCHS and WCHFS participants from tissue microarrays (TMAs) for multiplexed immune profiling.

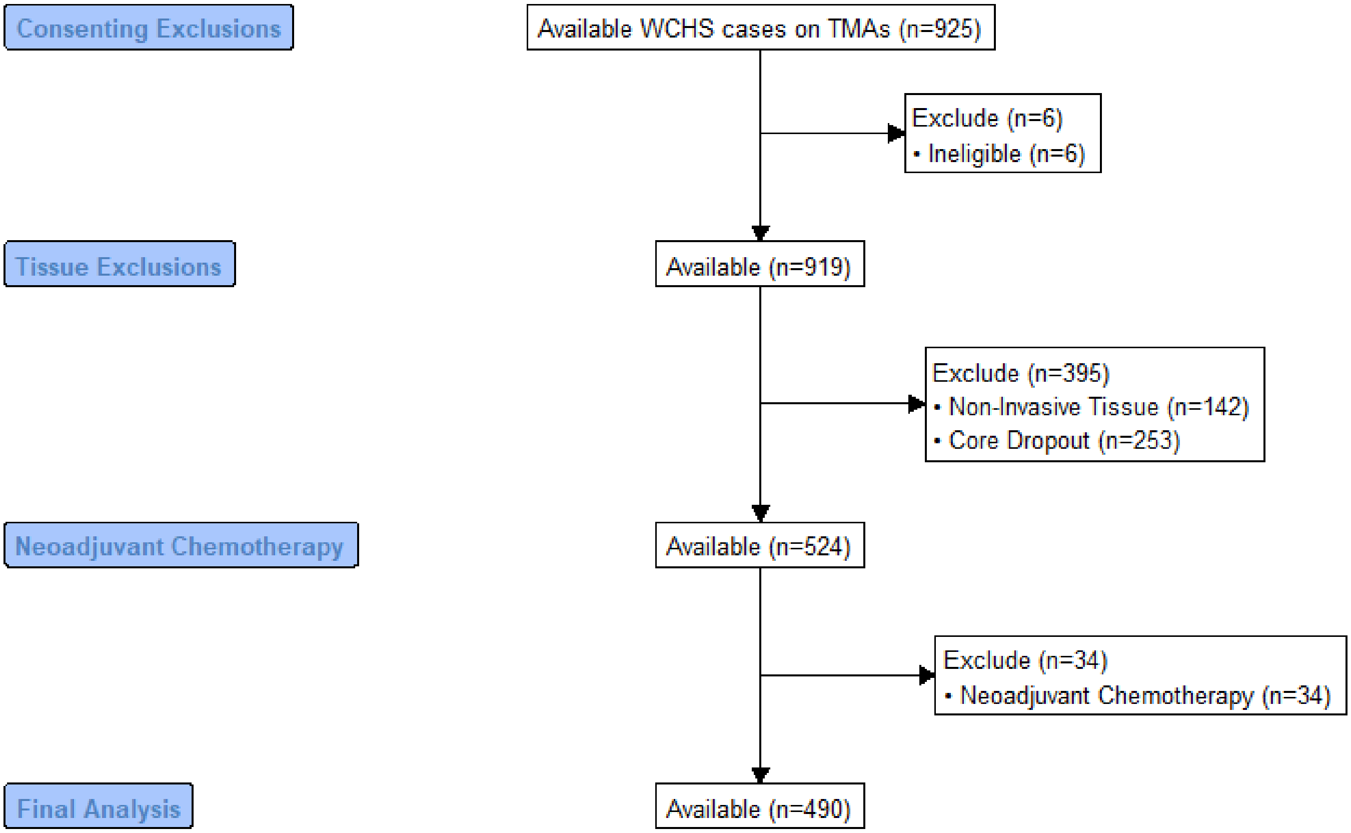

Figure S2. Cell density and cell percent correlation plots for multispectral imaging of T cell subsets.

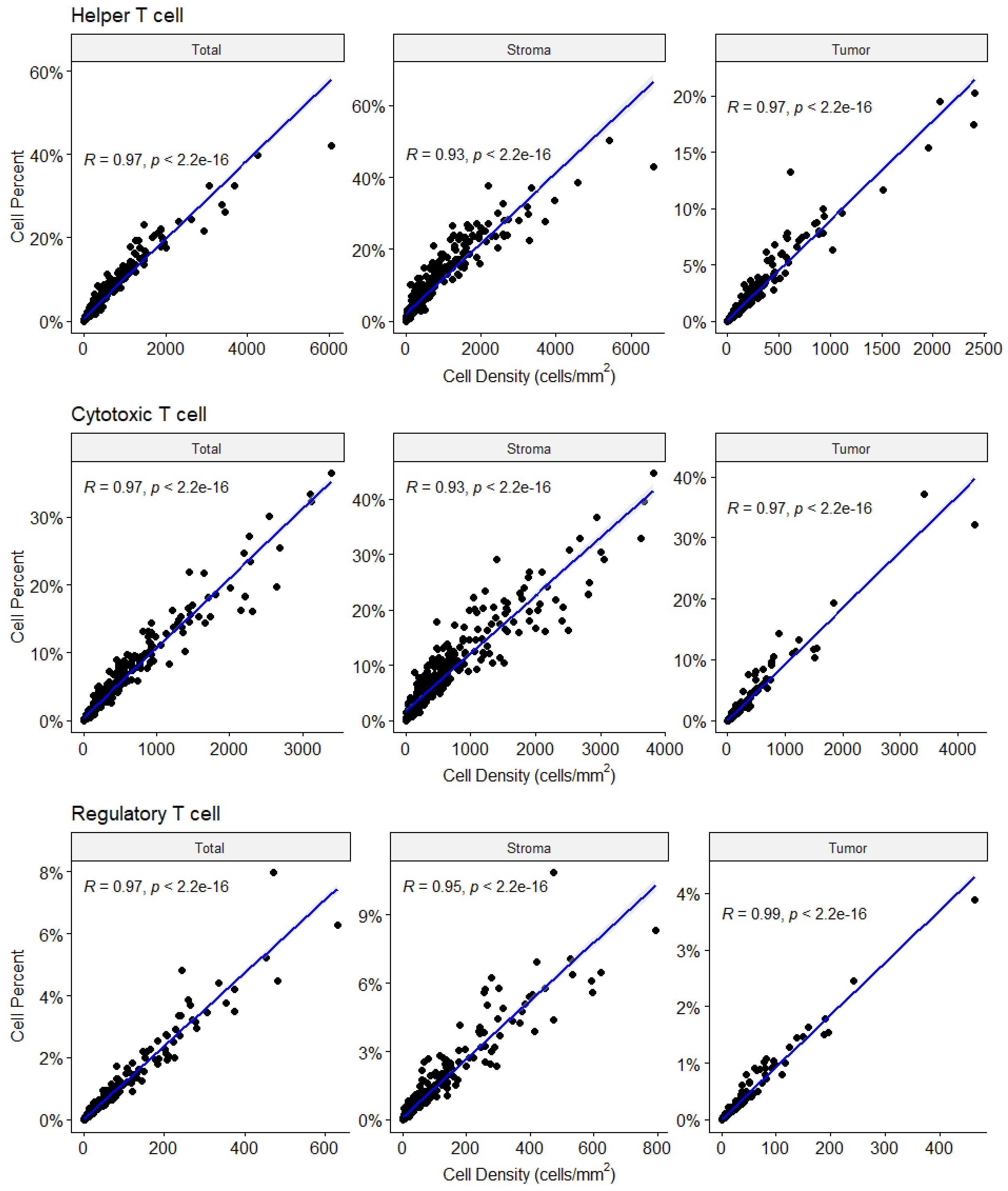

Figure S3. Correlation plots for multispectral IHC and conventional IHC for participants who had data available for both staining methodologies.

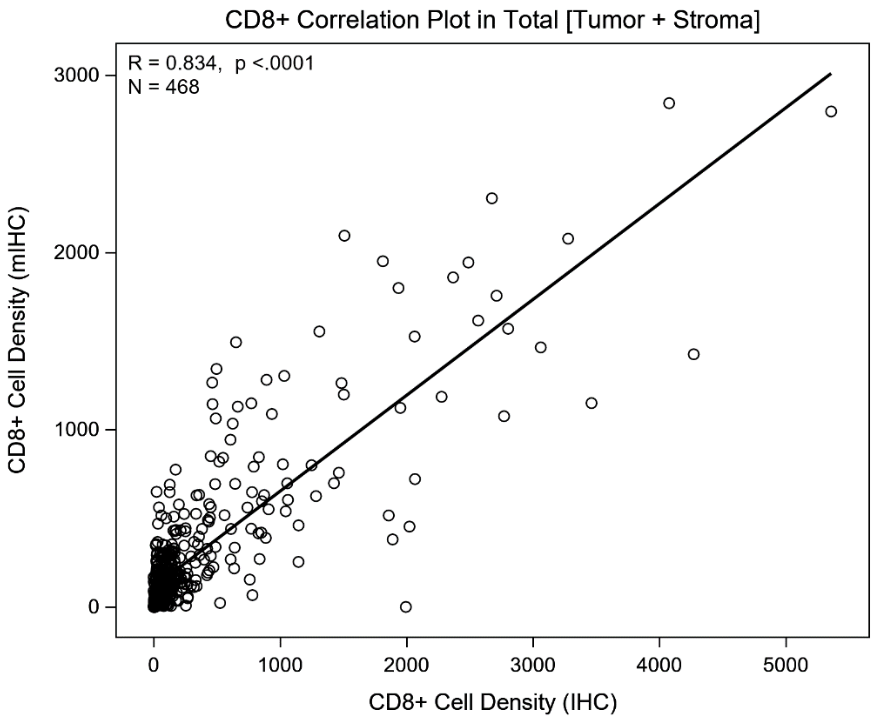

Figure S4. Marker densities across breast cancer tissue microarrays (TMAs). Twenty TMAs were stained (labelled A through T) in a single batch. Marker variability within TMA slide was higher than variability between TMA slides

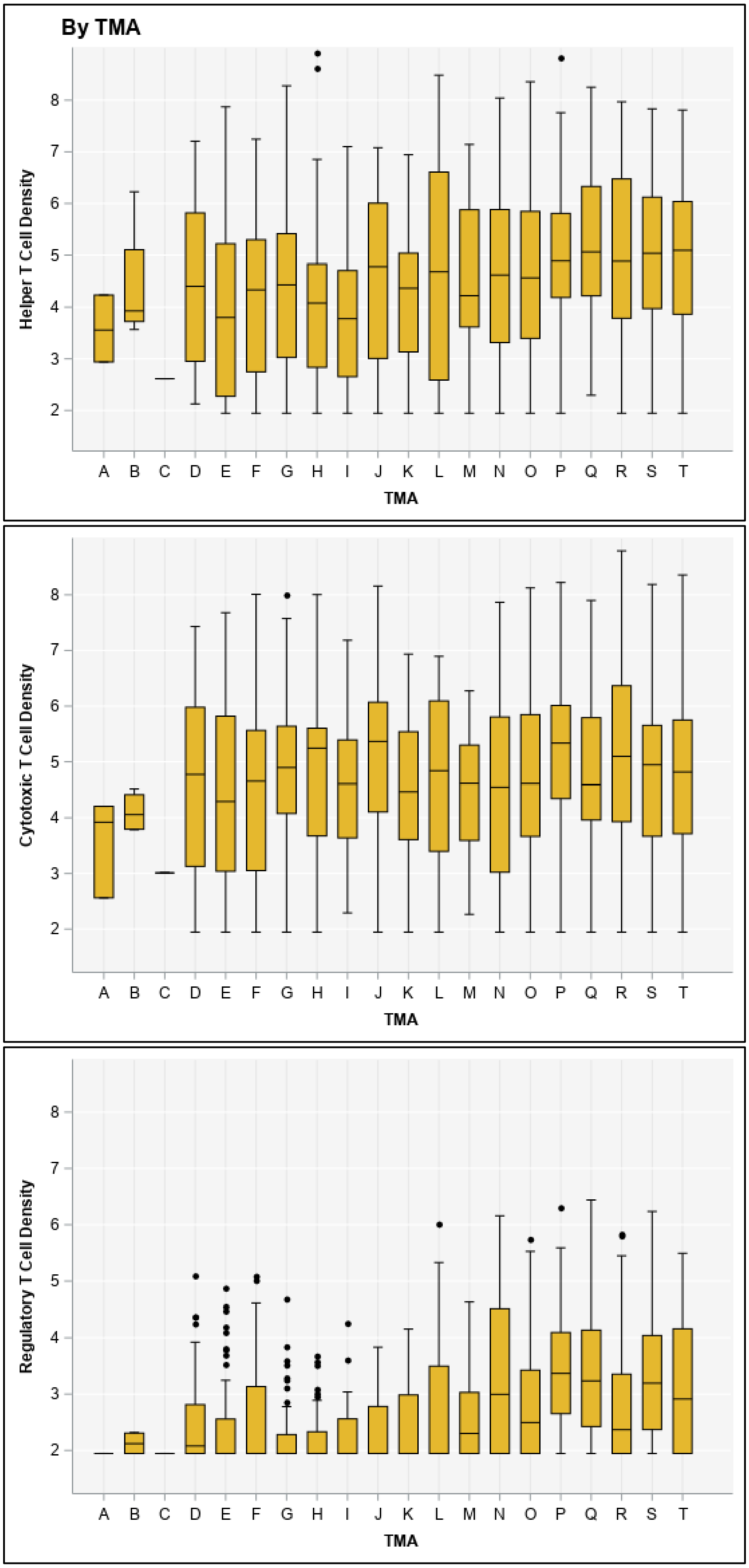

Figure S5. A rare population of CD8^{+}FOXP3^{+} immune cells was observed in 93 patient tumors. Composite fluorescent images are shown from four representative samples. Markers are DAPI (blue), CD4 (yellow), CD8 (magenta), ER (cyan), FOXP3 (orange), and pan-cytokeratin (red).

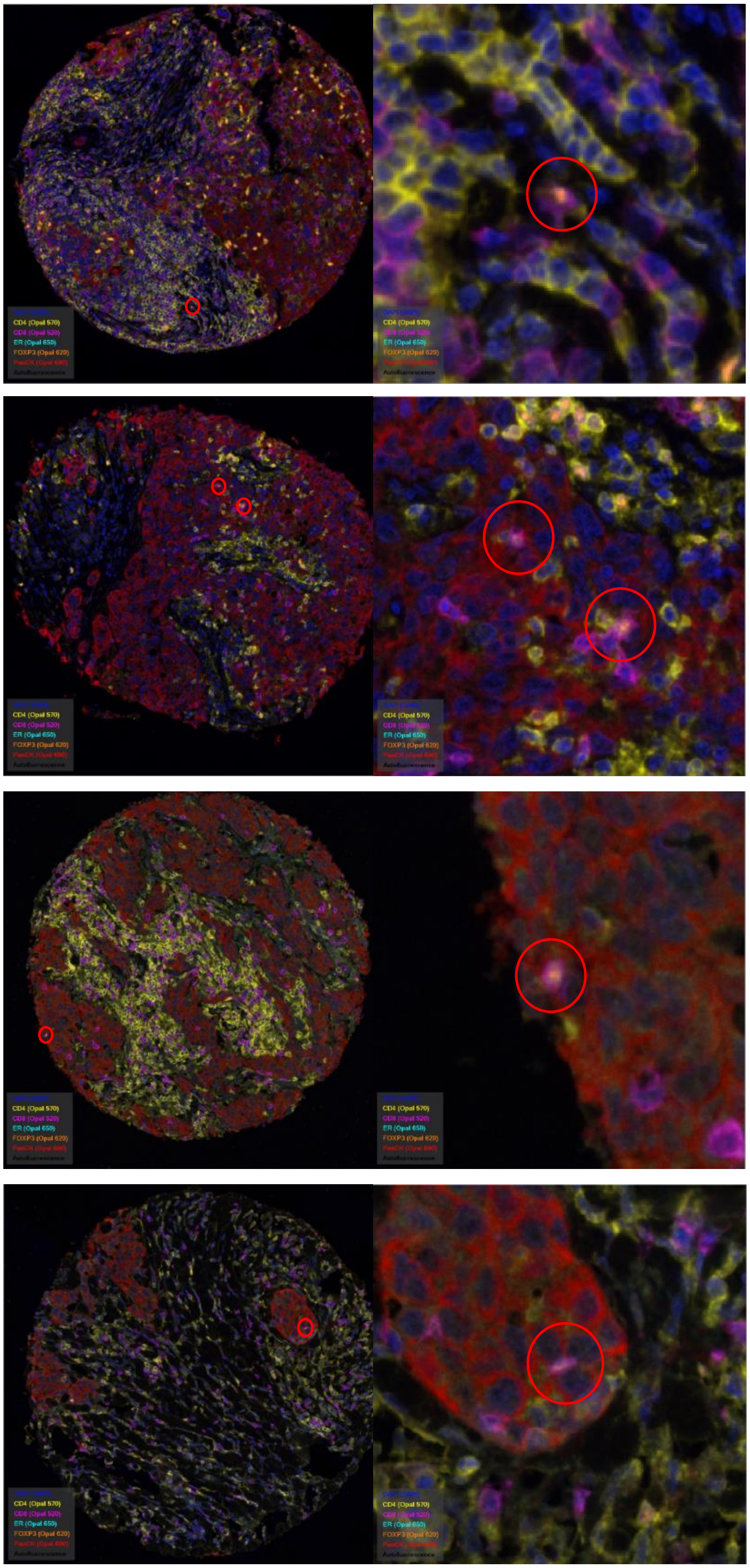

Patient 1

Patient 2

Patient 3

Patient 4
